# Cocaine Enhances HIV-1 Replication in CD4+ T Cells by Down-Regulating MiR-125b

**DOI:** 10.1371/journal.pone.0051387

**Published:** 2012-12-12

**Authors:** Chinmay K. Mantri, Jui Pandhare Dash, Jyoti Velamarti Mantri, Chandravanu C. V. Dash

**Affiliations:** The Laboratory of Retrovirology and Epigenetics, Center For AIDS Health Disparities Research, Vanderbilt-Meharry Center for AIDS Research, Department of Biochemistry and Cancer Biology, Meharry Medical College, Nashville, Tennessee, United States of America; National Institute of Health, United States of America

## Abstract

The main objective of this study was to examine effects of cocaine on HIV-1 replication in primary CD4+ T cells. Cocaine a commonly used drug among HIV-1 positive individuals serves as a cofactor for HIV-1 infection and progression to acquired immunodeficiency syndrome (AIDS). Accumulating evidence suggest that cocaine increases HIV-1 replication in cell cultures, peripheral blood mononuclear cells (PBMCs) and animal models. Intriguingly, there are no studies on cocaine-induced alterations in HIV-1 replication in primary CD4+ T cells that serve as the main targets for HIV-1 replication *in vivo*. In this report, we demonstrate cocaine-induced enhancement of HIV-1 replication in primary CD4+ T cells isolated from human PBMCs. To decipher a potential mechanism, we examined whether cocaine targets the innate antiviral immunity of CD4+ T cells mediated by cellular microRNAs (miRNAs). This is because recently a network of anti-HIV miRNAs in CD4+ T cells is highlighted to suppress viral replication. Our genome wide miRNA expression analysis indicated downregulation of several anti-HIV miRNAs (miR-28, miR-125b, miR-150, miR-223, and miR-382) in cocaine treated CD4+ T cells. However, our real-time quantitative PCR analysis revealed significant downregulation of miR-125b only. Our results illustrated that miR-125b knockdown enhances HIV-1 replication, whereas overexpression of miR-125b decreases HIV-1 replication in these cells. Therefore, we believe miR-125b is a key player for the cocaine induced enhancement of HIV-1 replication in CD4+ T cells. Since, miR-125b targets the 3′ UTR regions of HIV-1 transcripts and inhibits viral protein translation, our data suggest modulation of post entry steps of HIV-1 by cocaine. Given that a plethora of studies suggest that cocaine regulates HIV entry, our results implicate a potentially novel mechanism by which cocaine can increase viral replication in CD4+ T cells.

## Introduction

Illicit drug use remains the second most frequent mode of acquisition of HIV and drugs such as amphetamines, cocaine, marijuana, and opiates serve as cofactors for susceptibility to HIV infection and disease progression [1]–[8]. Cocaine, a commonly used drug among HIV-1 positive individuals, serves as a cofactor for HIV-1 infection and progression to acquired immunodeficiency syndrome (AIDS) [9]–[13]. Epidemiological studies suggest that HIV positive cocaine users have lower CD4+ T cell counts, increased risk of diseases progression and AIDS-related death [11]–[13]. *In vitro* cocaine increases HIV-1 replication in human peripheral blood mononuclear cells (PBMCs) and enhances viral load in humanized mice [14]–[16]. Although, cocaine is known to have immunomodulatory function [17]–[19], the underlying mechanism by which cocaine increases HIV-1 replication remains unclear. It has been proposed that cocaine increases HIV-1 infection/replication by inhibiting HIV-1 protective chemokines and/or upregulating the HIV-1 entry co-receptor [20]–[21]. For example, members of the β-chemokine family that bind to CCR5 such as regulated upon-activation T expressed and secreted (RANTES), macrophage inflammatory protein 1a (MIP-1a), and MIP-1b have been demonstrated to inhibit entry of certain HIV-1 strains [22]. In addition, proteomics analysis of cocaine treated PBMCs isolated from HIV-positive donors suggests that cocaine differentially regulates expression of several key host proteins that may influence HIV-1 replication [23]. Since these studies were conducted using cell culture models or the mixed cell populations of PBMCs, there are no reports on primary CD4+ T cells. Given that CD4+ T cells are the main targets for HIV-1 infection and replication *in vivo* [24], it is critical to evaluate whether cocaine enhances HIV-1 replication in primary CD4+ T cells.

MicroRNAs (miRNAs) are noncoding single-stranded small RNAs that bind to the 3′ untranslated region (UTR) of target mRNA and post-transcriptionally regulate gene expression via degradation of specific mRNAs and/or repression of their translation [25]. Therefore, cellular miRNAs play critical roles for the normal function and diseased state of cells [25]. MiR-125 is a highly conserved miRNA from nematode to humans and has recently emerged as a key regulator of cell survival and differentiation [26]. In addition miR-125b has been shown to be dysregulated in multiple malignancies [26]. miR-125b is the ortholog of lin-4 in *Caenorhaditis elegans* and is transcribed from two loci located on chromosomes 11q23 (hsa-miR-125b-1) and 21q21 (hsa-miR-125b-2) [27]. miR-125b has been reported to target many key proteins that regulate apoptosis, innate immunity, inflammation and hematopoietic differentiation [26]. Recently, miR-125b has been demonstrated to regulate a network of genes in CD4+ T cells that are critical for its differentiation [28]. Intriguingly, miR-125b also belongs to the network of cellular anti-HIV miRNAs that suppress viral replication [29]–[30]. These anti-HIV miRNAs target the 3′ UTR regions of HIV-1 transcripts and inhibit HIV-1 replication. It has been proposed that miR-125b and other anti-HIV-1 miRNAs may be responsible for inducing latency in naïve CD4+ T cells [29]. Very recently, it has also been suggested that downregulation of miR-125b in the PBMCs of HIV-1 infected individuals may lead to viremia [31].

Since CD4+ T cells serve as primary targets for HIV-1 infection and replication [24], in this study we have examined whether cocaine enhances HIV-1 replication in CD4+ T cells. Using primary CD4+ T cells isolated from human PBMCs, we illustrate cocaine-induced increase in HIV-1 replication in these cells. In an attempt to decipher the mechanism by which cocaine enhances HIV-1 replication, we examined whether cocaine targets the anti-HIV-1 miRNAs in CD4+ T cells. The rationale is derived from the accumulating evidence that cellular miRNAs confer antiviral innate immunity and may negatively regulate HIV-1 replication [29]–[30], [32]–[33]. Therefore, we carried out genome wide miRNA expression analysis to investigate whether cocaine modulates cellular anti-HIV-1 miRNA expression in primary CD4+ T cells. Our genome wide miRNA results indicated downregulation of several anti-HIV-1 miRNAs. However, our real time PCR analysis demonstrated substantial downregulation of miR-125b in uninfected and infected activated CD4+ T cells. This cocaine induced downregulation of miR-125b resulted in increased HIV-1 replication in CD4+ T cells. This was confirmed by knock-down and overexpression studies of miR-125b. Furthermore, our promoter reporter assay revealed that cocaine treatment resulted in downregulation of miR-125b promoter activity. Given that miR-125b inhibits HIV-1 protein translation, the data presented in this report demonstrate a role of post entry steps of HIV-1 by which cocaine enhances HIV-1 replication. Therefore, our results implicate a potentially novel mechanism by which cocaine can increase viral replication in HIV- 1 positive drug addicts.

## Materials and Methods

### Healthy Donors, Isolation of PBMCs, Purification of CD4+ T Cells and Cell Culture

Human blood was purchased from the New York Blood Center as per the Meharry Medical College IRB from 12 healthy donors. For PBMC isolation fresh human blood was diluted 1∶2 with Phosphate Buffered Saline (PBS). Subsequently, 25 ml of diluted blood was overlaid on 12.5 ml of Ficoll-Paque™Premium reagent (GE) in a 50 ml conical tube and centrifuged at 750×g without break for 20 minutes at 20°C. Thereafter, the interphase cells (PBMCs) were transferred carefully to a new 50 ml tube and PBS was added to make up to 50 ml. Subsequently, the PBMCs were centrifuged several times and washed with PBS to remove unwanted cell types. The resulting cell pellet was resuspended in PBS followed by counting and viability determination by trypan blue exclusion. CD4+ T cells were isolated by negative selection as per the standard protocol described in CD4^+^ T cell Isolation Kit II (Miltenyi Biotec). The purity of isolated CD4+ T cells was checked by Flow Cytometry (see below). The CD4+ T cells were activated by PHA (5 mg/ml) for 48 h, and maintained with interleukin-2 (20 U/ml; Sigma). SupT1, a T cell line, was obtained from American Type Culture Collection (ATCC) and maintained in complete RPMI (cRPMI) that contains RPMI with 10% fetal bovine serum (FBS) and antibiotics**.** ACH-2 and TZM-bl cells were obtained from NIH AIDS Research and Reference Reagent Program, Division of AIDS, NIAID, NIH: ACH-2 cells from Dr. Thomas Folks [34]–[35] and TZM-bl cells from Dr. John C. Kappes, Dr. Xiaoyun Wu and Tranzyme [36].

### Cocaine Treatment

Cocaine hydrochloride was obtained from the National Institute on Drug Abuse (NIDA) Drug Supply Program. We use 1 µM cocaine in our studies because of its relevance to human plasma levels of drug users that often range between 0.4–1.6 µM for intranasal administration [37]–[38].

### Microarray Analysis

Purified CD4+ T cells were treated with cocaine for 24 hours and total RNA was isolated with miRNeasy mini Kit (Qiagen). RNA processing, microarray fabrication, hybridization, data acquisition and analysis were performed at the Vanderbilt University Genomics Core. Briefly**,** 1 µg of total RNA (control and treated, in triplicates) was used for miRNA expression profiling using the miRCURY LNA microRNA Arrays version 11.0 (Exiqon, DK). Microarray data acquisition was done with GenePix Pro v. 6.1 acquisition & Analysis Software. miRNA microarray results were analyzed using an ANOVA statistical test.

### Real Time RT-PCR Analysis

HIV-1 infected and uninfected CD4+ T cells were treated with cocaine and total RNA was isolated by miRNeasy mini Kit (Qiagen). Real Time RT-PCR was carried out with the RNA using miRNA specific primers (Exiqon) in C1000 Touch™ Thermal cycler (Bio-Rad). PCR results were analysed in Bio-Rad CFX manager software. miRNA expression levels were normalized to 5s-rRNA and GAPDH expression.

### Infectious and Pseudotyped Virus Production

For infectious HIV-1, we use the supernatant of chronically infected ACH-2 cells (with HIV-1 LAI isolate). ACH-2 cells were plated (5×10^5^ cells/ml) in cRPMI media overnight and were activated with PMA and TNF-α for 1 hr. Thereafter, the cells were washed twice with PBS and incubated overnight at 37°C with fresh cRPMI media. The virus containing supernatant was collected by centrifugation and filtering through a 0.45 µM filter. We generated VSV-G pseudotyped HIV-1 by transfecting the pNL4.3 molecular clone of HIV-1 and pVSV-G into 293T cells using Lipofectamine (Invitrogen). After 48 hr, culture supernatant containing the VSV-G pseudotyped virions was collected, centrifuged and filtered through a 0.45 µM membrane. pLVSV-G and pNL4.3dE.dV-Luc (a gift of Dr. Vineet KewalRamani, NCI/NIH) and pNL4.3dE.dV-RFP (NIH AIDS Reagent Program) were transfected to 293T cells and the virus was collected after 48–72 hr. Concentration of the virus was determined by p24 ELISA assay and infectivity was measured by luciferase reporter assay using TZM-bl cells which harbors a firefly luciferase reporter gene under the control of HIV-1 promoter.

### Infection

PHA Activated CD4+ T cells (1×10^6^ cells) were infected with HIV-1 LAI (∼MOI of 5) or VSV-G pseudotyped virus (MOI ∼ 0.1) by spinoculation in the presence of polybrene (Sigma) and were cultured (4×10^5^ cells/ml) for 1–2 weeks in the presence or absence of cocaine. Productive infection was measured by FACS by detecting intracellular HIV-1 p24 protein. TZM-bl cells that express CD4 and CXCR4/CCR5 receptors and harbor an integrated copy of firefly luciferase gene under the control of HIV-1 LTR promoter, were infected in the presence of polybrene (Sigma) for 6 hours. Thereafter, cells were washed with PBS and were incubated at 37°C. After 48–72 hrs, TZM-bl cells were washed, lysed and luciferase activity was measured using a Synergy HT Multi-Mode Microplate Reader.

### Knockdown and Overexpression of miR-125b in CD4+ T Cells

miR-125b inhibitors and negative controls were purchased from Dharmacon (Lafayette, CO). 10–100 pmole of anti-miRNAs or negative control was transfected to SupT1 cells using Neon Transfection System (Invitrogen). Cells were recovered in pre-warmed antibiotic-free RPMI medium and incubated for 3 h at 37°C/5% CO_2_. Thereafter, these cells were infected to determine the effect of miR-125b on HIV-1 replication.

miR-125b mimic and negative controls were purchased from Dharmacon (Lafayette, CO). 100–200 pmole of miR-125b mimic or negative control was transfected to CEM cells (a gift from Richard D’Aquila, Vanderbilt) using Neon Transfection System (Invitrogen). Cells were recovered in pre-warmed antibiotic-free RPMI medium and incubated for 3 h at 37°C/5% CO_2_. Thereafter, these cells were infected to determine the effect of miR-125b on HIV-1 replication.

### miR-125b Complementation Assay

SupT1 cells (5×10^4^) were infected with VSV-G pseudotyped HIV (MOI ∼ 0.1) by spinoculation in the presence of polybrene and the infected cells were grown in presence of cocaine for 36 hr. Thereafter, cells were transfected with 100 pmole of miR-125b mimic or negative control using Neon Transfection System (Invitrogen) and cells were recovered in pre-warmed antibiotic-free RPMI medium. After 2hr incubation cells were transferred to complete RPMI medium and cultured for another 24hr at 37°C/5% CO_2_. Thereafter, these cells were harvested for Luciferase assay by Luciferase Assay System (Promega). Luciferase activity was measured using Synergy HT Multi-Mode Microplate Reader (BioTek) and normalized to total protein content of the lysate.

### miR-125b Promoter Transcription Assay

pGL3 Basic (Promega) and pGL3-miR-125b-1 reporter (a kind gift from Dr. Xian Ming Chen, Creighton University Medical Center, Omaha, NE ) containing miR-125b promoter was transfected into 293T cells using Lipofectamine (Invitrogen) for 24 hr. Luciferase assay was carried out after treating the cells with 0.1 µM and 1 µM cocaine for 4 hr. Luciferase activity was measured using Synergy HT Multi-Mode Microplate Reader (BioTek) and normalized to total protein content of the lysate. The luciferase activity of miR-125b construct was compared with that of the promoter less pGL3 basic vector.

### FACS Analysis

To check the purity of isolated CD4+ T cells, cells were washed twice with PBS containing 0.25% EDTA and 0.5% BSA and incubated with anti-CD4 antibodies for 15 minute at 4°C in the same buffer. After staining cells were washed two times with the same buffer. For intracellular p24 staining, cells were first fixed with 4% formaldehyde solution and permeabilised for 20 minutes in BD Cytofix/Cytoperm™ Plus Permeabilization Buffer. After permeabilization, cells were incubated with 5 µl of anti-p24 antibody or IgG isotype control antibody for 20 min at 4°C. Then cells were washed twice with BD Cytofix/Cytoperm™ Plus Permeabilization Buffer and resuspended in the same buffer. Cells were analyzed on a BD FACSCalibur™ platform and data analysis was done with Cell Quest Pro (BD) or FlowJo (Tristar) software. FITC–anti-CD4; FITC- and PE- anti-CD25; and FITC- IgG antibodies were obtained from Miltenyi Biotec. Anti-p24-FITC antibody was obtained from Beckman Coulter.

## Results

### Cocaine Enhances HIV-1 Replication in Primary CD4+ T Cells

**Figure 1:**
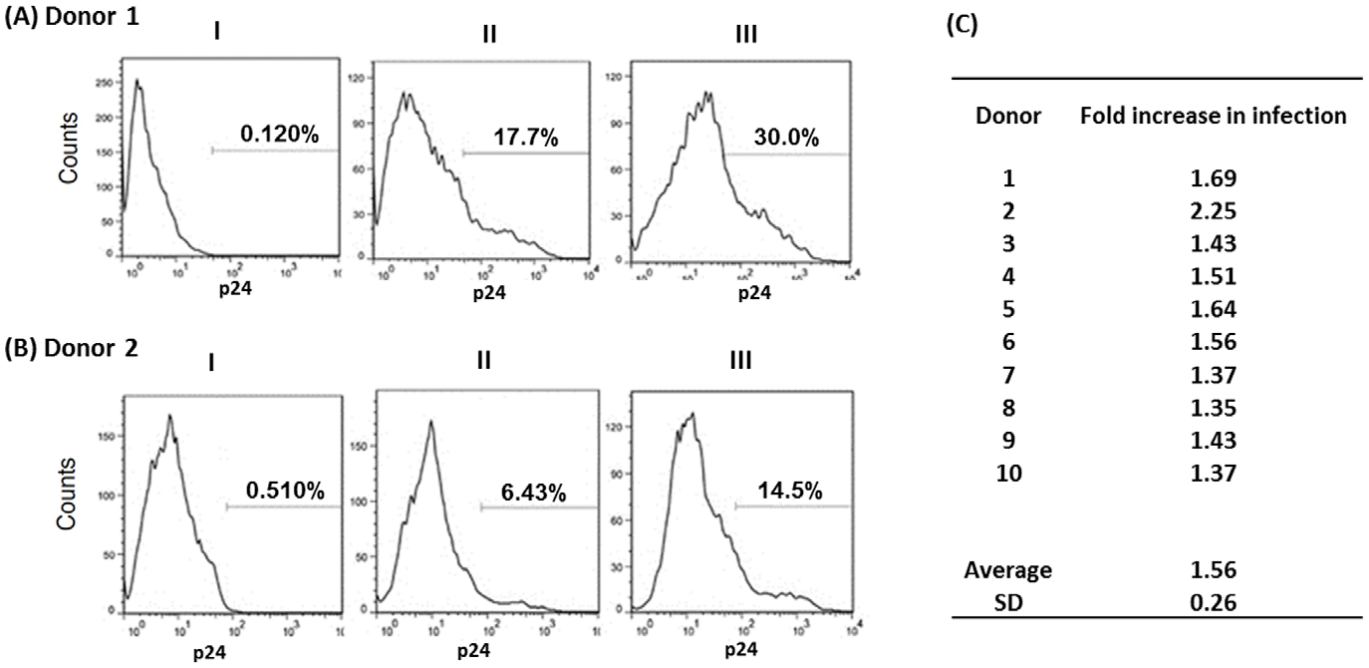
Cocaine-enhances HIV-1 replication in primary CD4+ T cells. Primary CD4+ T cells were isolated by negative selection from human PBMCs. After isolation, these cells were activated by PHA for 48–72 hrs and infected with infectious HIV-1 LAI by spinoculation and cultured in the presence or absence of cocaine. Productive infection was measured by detecting viral p24 protein 7 days post infection by FACS. (A–B) Representative data from two donors. Panels I: p24 staining of uninfected cells. Panels II: p24 staining of HIV-1 infected cells in the absence cocaine. Panels III: p24 staining of HIV-1 infected cells in the presence of cocaine. (C) Data from ten donors with fold increase in HIV-1 replication in presence of cocaine. Cocaine treatment significantly increased the percentage of cells expressing viral p24 protein in these cells. Data are representative of three independent experiments comprising nine different donors.

It has been described that cocaine enhances HIV-1 replication in PBMCs and animal models [14]–[16]. Surprising, there are no reports on the effects of cocaine on HIV-1 replication in CD4+ T cells that are the primary targets of HIV-1. To test this, we isolated PBMCs from peripheral blood of normal human donors and purified CD4+ T cells by negative selection. These primary CD4+ T cells were activated and infected with infectious HIV-1 and treated with cocaine after infection. Productive infection was examined after 7 days by detecting intracellular viral p24 antigen by FACS. Our FACS analysis illustrate that cocaine enhances HIV-1 replication in primary CD4+ T cells (Fig. 1), since significantly higher percentage of cells expressed viral p24 in the presence of cocaine in comparison to in the untreated cells. Importantly, this enhancement is not dependent on donors used as reflected in the data presented in Fig. 1C that illustrate a consistent increase in HIV-1 replication in the presence of cocaine. To our knowledge these data sets provide the first evidence on cocaine-induced enhancement of HIV-1 replication in primary CD4+ T cells.

### Cocaine Enhances HIV-1 Replication by Modulating Post Entry Steps

**Figure 2:**
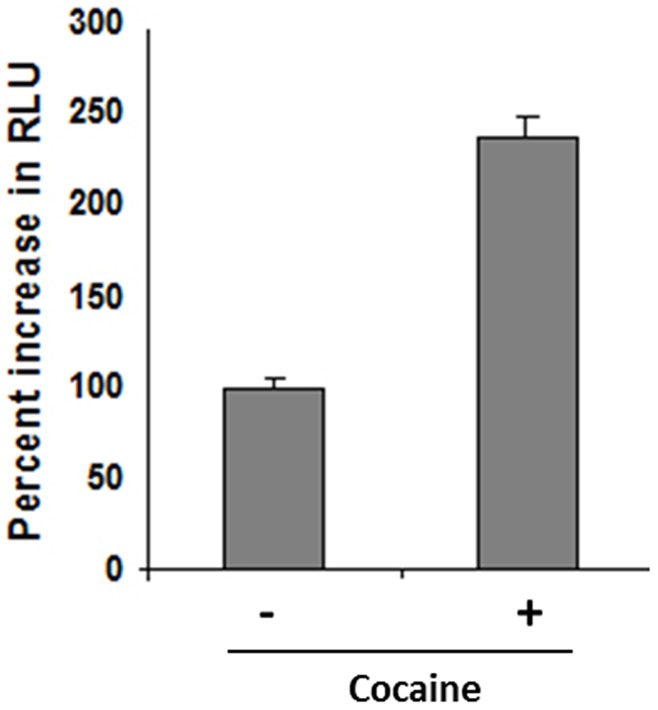
Cocaine-enhances HIV-1 replication by targeting post entry steps. To examine whether cocaine targets post entry steps of HIV-1 life cycle, we abrogated cellular receptor requirement for viral entry by pseudytyping HIV-1 virions with VSV-G envelop. Thereafter, TZM-bl cells (that harbor an integrated copy of luciferase reporter gene under the control of HIV-1 LTR promoter) were infected with VSV-G pseudotyped HIV-1 virions in the presence and absence of cocaine. Productive infection was measured after 48–72 hrs by HIV-1 LTR driven luciferase expression. Data are representative of three independent experiments conducted in triplicates.

HIV-1 entry is mediated by CD4 receptor and CXCR4/CCR5 co-receptors [39]–[41]. It has been proposed that cocaine increases HIV-1 replication by either upregulating these entry co-receptor or inhibiting HIV-1 suppressing chemokines on the target cell [20]–[21]. In our experiment described in Fig. 1 however, cocaine was added to the cells post-infection. Therefore we hypothesized that cocaine may modulate viral post entry steps. To examine this, we abrogated entry receptor (CD4 and CXCR4/CCR5) requirement by pseudotyping HIV-1 virions with the vesicular stomatitis virus glycoprotein (VSV-G). VSV-G pseudotyped virions are known to mediate viral entry through endocytosis [42]. These pseudotyped virions were used to infect TZM-bl cells that harbor an integrated copy of firefly luciferase gene under the control of HIV-1 LTR promoter. After 48–72 hrs, the cells were lysed and luciferase activity was measured. Results from this experiment demonstrate increased HIV-1 transcription as measured by luciferase activity in the presence of cocaine (Fig. 2). Since pseudotyped HIV-1 does not depend on CD4 and CXCR4/CCR5 for entry, our data in Fig. 2 strongly suggest that cocaine modulates viral post entry steps to enhance HIV-1 replication. Therefore, we propose that cocaine impacts both the viral entry and post entry steps to enhance HIV-1 replication.

### Cocaine Down-regulates miR-125b in CD4+ T Cells

**Figure 3:**
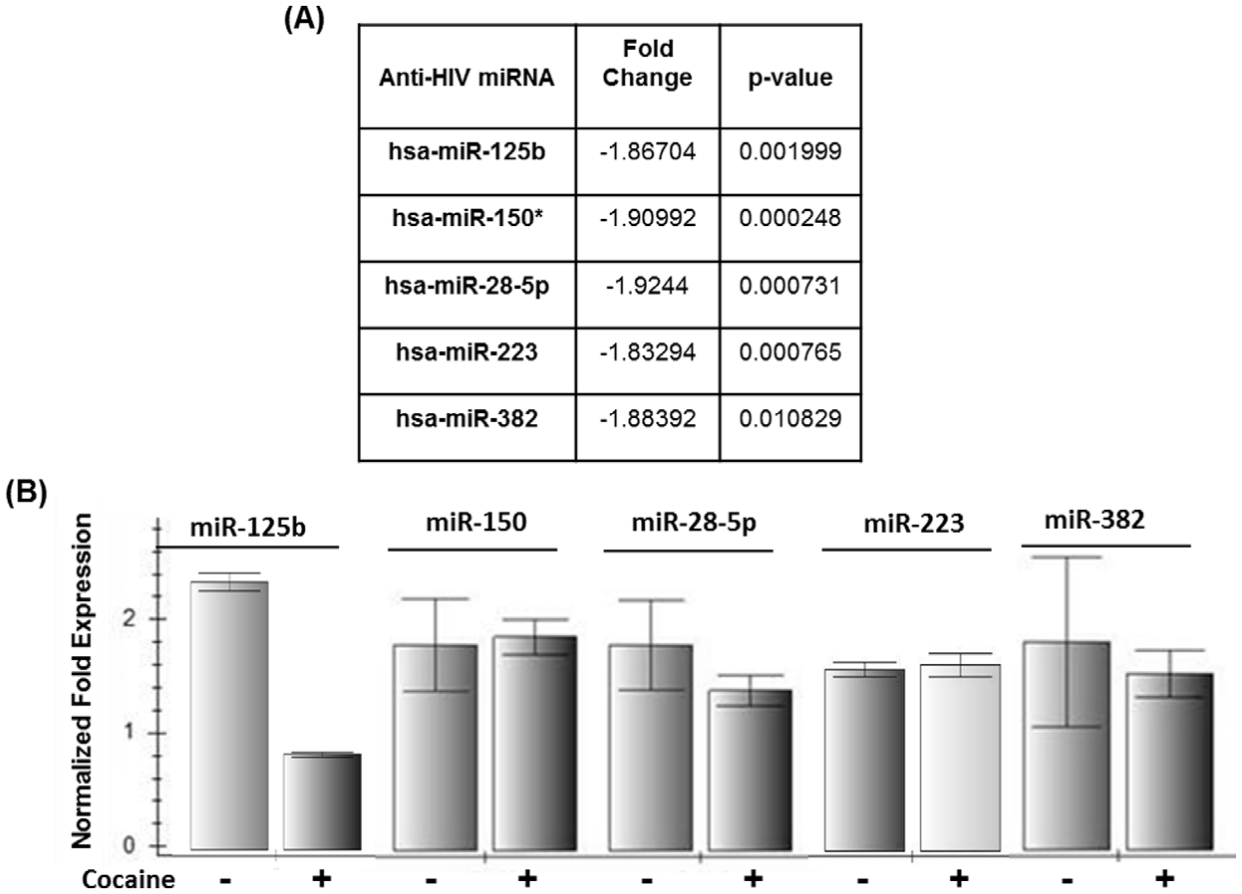
Cocaine downregulates anti-HIV-1 miRNAs in CD4+ T cells. Activated primary CD4+ T cells were treated with cocaine for 24 hrs and expression of cellular miRNAs were analyzed by microarray and compared with untreated cells. The expressions of cellular anti-HIV-1 miRNAs with highly significant *p* values were considered. The table shows that cocaine treatment downregulated a family of anti-HIV-1 miRNAs including miR-125b, miR-150, miR-28p, miR-223, and miR-382 in CD4+ T cells. (**B**) To confirm our microarray data, we carried out real time PCR analysis using RNA isolated from cocaine treated CD4+ T cells and compared expression of these miRNAs with untreated cells. miRNA expression levels were determined by miRNA specific primers and normalized to 5s-rRNA and GAPDH expression. miR-125b was consistently downregulated upon cocaine treatment, whereas expression of other miRNAs (miR-26, miR-150, miR-223, miR-122, and miR-296) were not significantly affected. Data are representative of three independent experiments comprising six to nine different donors.

In an attempt of decipher the specific steps of viral post entry that is targeted by cocaine, we examined whether cocaine modulates the anti-HIV-1 miRNAs in CD4+ T cells. This is because a family of anti-HIV cellular miRNAs has been recently described in CD4+ T cells that confer antiviral innate immunity [29]. Therefore, we carried out genome wide miRNA analysis to investigate whether cocaine modulates cellular anti-HIV miRNA expression in primary CD4+ T cells. Our microarray data revealed downregulation of an array of cellular miRNAs (data not shown) including anti-HIV-1 miRNAs (miR-125b, miR-150, miR-28-5p, miR-223, and miR-382) in cocaine treated cells (Fig. 3A). It has been previously described that these anti-HIV-1 miRNAs target the 3′ UTR regions of HIV-1 transcripts and inhibit viral translation [29]–[30]. Therefore, we carried out real time PCR analysis to confirm our microarray data and the results are presented in Fig. 3B. Our data illustrate that cocaine treatment significantly downregulated “miR-125b” expression in these cells. Other anti-HIV-1 miRNAs that were downregulated in microarray analysis (Fig. 3A) were not downregulated in our real time PCR experiments. Quantitative real-time PCR (qPCR) is commonly used to validate gene expression results obtained from microarray analysis. However, microarray and qPCR data often result in disagreement and both techniques suffer from pitfalls [43]. For example, the quality of gene expression data obtained from microarrays can vary greatly with platform and procedures used [43]. Therefore, the pairing of microarray and qPCR is common in gene expression studies that produce more reliable results [43]. Given that both real time PCR and microarray analysis demonstrated downregulation of miR-125b, we believe the effects of cocaine on miR-125b are real. Therefore, we tested a role of miR-125b in HIV-1 replication.

### Cocaine-induced Down-regulation of miR-125b Enhances HIV-1 Replication in CD4+ T Cells

**Figure 4:**
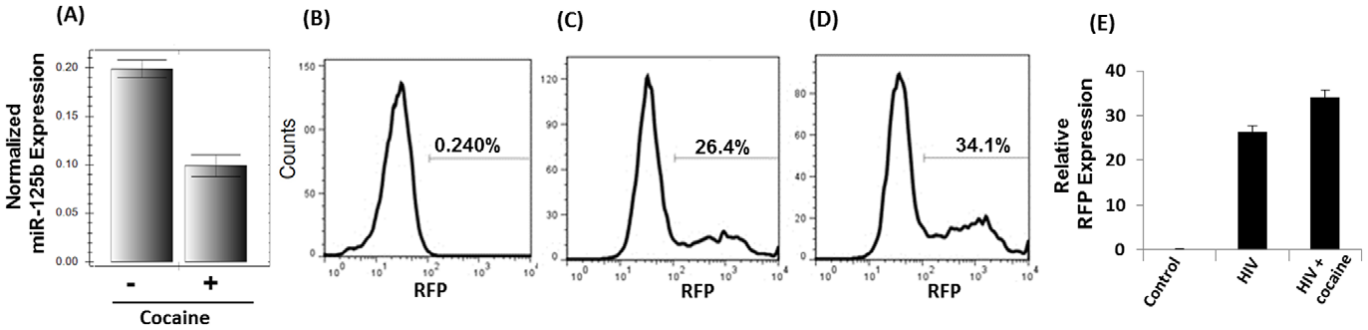
Cocaine induced downregulation of miR-125b enhances HIV-1 replication. We carried out single cycle replication assay to evaluate modulation of HIV-1 replication by cocaine. For this experiment we used a T cell line SupT1 that expresses miR-125b. (**A**) Cocaine treatment substantially downregulated miR-125b in SupT1 cells as determined by real time PCR. Pseudotyped HIV-1 virions with RFP reporter were used to infect SupT1 cells and HIV-1 replication was measured by detecting RFP expression by FACS 48–72 hr post infection. (**B**) RFP expression of uninfected cells. HIV-1 replication was measured in the absence (**C**) and presence (**D**) of cocaine. (**E**) Relative RFP expression from three independent experiments. Cocaine treatment resulted in increased RFP expression implying enhanced HIV-1 replication. Data are representative of three independent experiments conducted in triplicates.

Since miR-125b was significantly downregulated in cocaine treated CD4+ T cells both by microarray and real time PCR analysis, we evaluated whether downregulation of miR-125b can enhance HIV-1 replication. To achieve this we used the T cell line SupT1 since it supports HIV-1 replication. Most importantly, SupT1 cells express miR-125b and cocaine treatment induces substantial downregulation of miR-125b expression (Fig. 4A). Therefore, we tested whether cocaine treatment can increase HIV-1 replication in SupT1 cells. We infected SupT1 cells with pseudotyped HIV-1 with a RFP reporter and analyzed productive infection by measuring RFP expression by FACS. Our results indicated that the percentage of cells expressing RFP is increased significantly when the cells were treated with cocaine (Fig. 4 C–D). Since this a single cycle replication assay, these results reaffirm that cocaine targets post entry steps of the viral life cycle to enhance HIV-1 replication.

### Knock-down of miR-125b Enhances HIV-1 Replication

**Figure 5:**
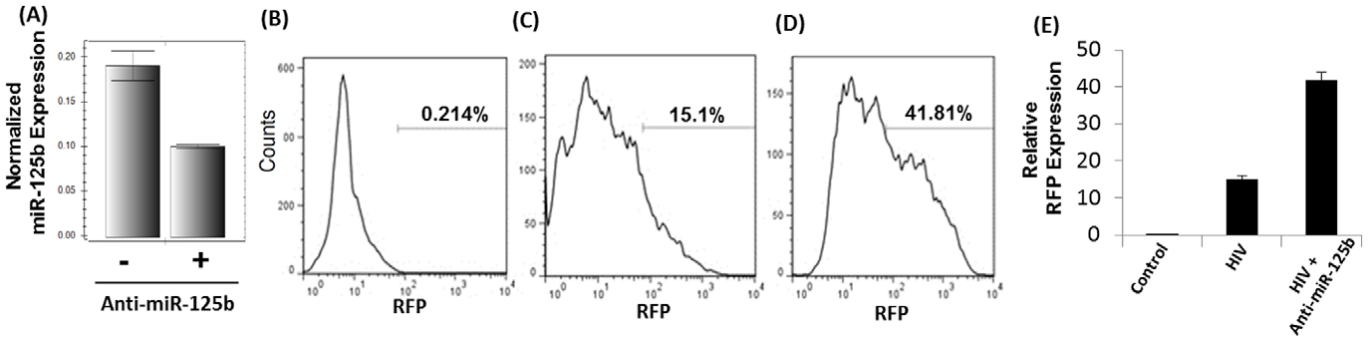
Knock-down of miR-125b enhances HIV-1 replication. (**A**) miR-125b knockdown experiments were conducted using anti-miR-125b and SupT1 cells. These cells were infected with VSV-G pseudotyped HIV-1-RFP reporter virus and infection was determined by FACS. Since cycle replication was determined by measuring intracellular RFP expression. (**B**) RFP expression of uninfected cells by FACS analysis. HIV-1 replication was measured in the absence (**C**) and presence (**D**) of anti-miR-125b. (**E**) Relative RFP expression from three independent experiments. miR-125b knock-down resulted in increased RFP expression implying miR-125b plays a critical role for cocaine-induced enhancement of HIV-1 replication. Data are representative of three independent experiments conducted in triplicates.

To evaluate whether cocaine targets miR-125b to enhance HIV-1 replication, we conducted knockdown experiments using anti-miR-125b and SupT1 cells. As illustrated in Fig. 5A, our knockdown experiments were designed to achieve the level of miR-125b expression to 50% that mimics cocaine-induced downregulation as presented in Fig. 3B and Fig. 4A. Subsequently, we infected these cells with VSV-G pseudotyped HIV-1-RFP reporter virus and determined infection by measuring RFP expression by FACS after 48–72 hrs. A comparative analysis of infection experiments illustrates that knock-down of miR-125b enhanced RFP expression in SupT1 cells from 15% to 41%. This enhancement demonstrates that miR-125b plays a critical role for cocaine-induced increase of HIV-1 replication in CD4+ T cells.

### Over-expression of miR-125b Inhibits HIV-1 Replication

**Figure 6:**
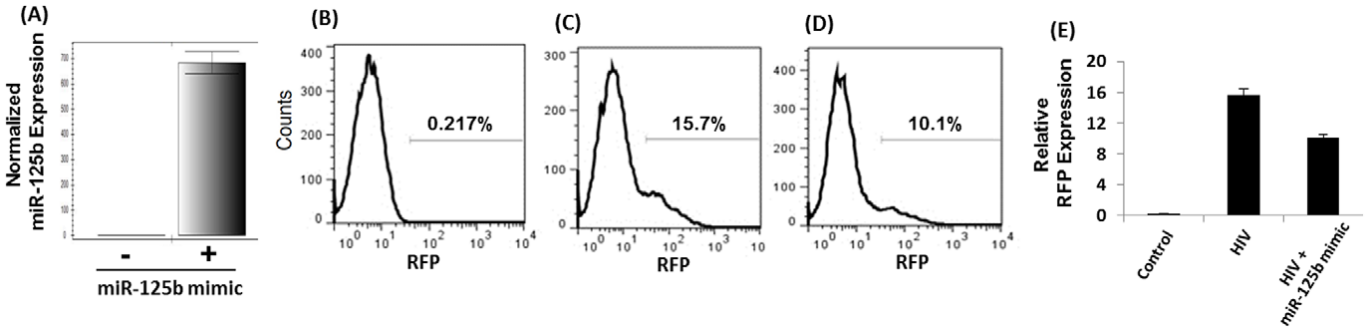
Over-expression of miR-125b inhibits HIV-1 replication. (**A**) miR-125b over-expression experiments were conducted using anti-miR-125b and CEM cells. These cells were infected with VSV-G pseudotyped HIV-1-RFP reporter virus and infection was determined by FACS. Since cycle replication was determined by measuring intracellular RFP expression. (**B**) RFP expression of uninfected cells. HIV-1 replication was measured in the absence (**C**) and presence (**D**) of miR-125b mimic. (**E**) Relative RFP expression from three independent experiments. miR-125b over-expression resulted in decreased RFP expression implying miR-125b modulates HIV-1 replication. Data are representative of three independent experiments conducted in triplicates.

To further validate a role of miR-125b in HIV-1 replication, we over-expressed miR-125b in CEM cells using miR-125b mimics. The rational for using CEM cells is that they express miR-125b at very low or undetectable levels and higher miR-125b levels are achieved by transfection of miR-125b mimic (Fig. 6A). Infection of these cells with VSV-G pseudotyped HIV-1 RFP resulted in reduction of RFP expression in comparison to control cells (Fig. 6B-E). These data sets further support our arguments for a role of miR-125b in inhibiting HIV-1 replication (Fig. 6B–E).

### Effect of Cocaine on HIV-1 Replication is Dependent on miR-125b Expression

**Figure 7:**
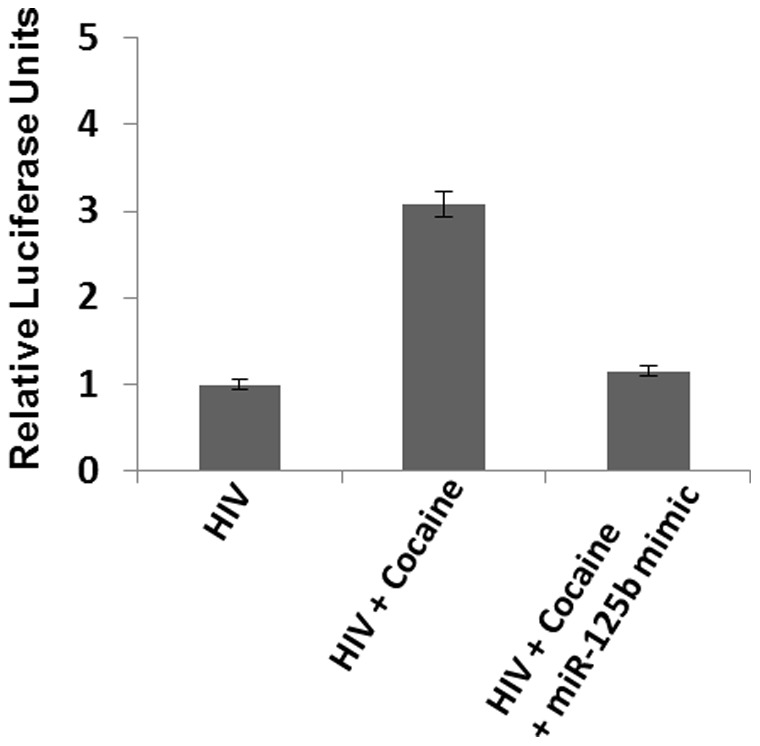
Effect of Cocaine on HIV-1 replication is dependent on miR-125b expression. SupT1 cells were infected with VSV-G pseudotyped HIV-1-Luciferase reporter virus and infection was determined by luciferase activity. For miR-125b complementation assay cocaine treated infected cells were transfected with miR-125b mimic. The increase in HIV-1 replication by cocaine was abrogated by miR-125b mimic expression. Data are representative of three independent experiments conducted in triplicates.

To evaluate whether cocaine-induced downregulation of miR-125b directly contributes to increased HIV-1 replication, we carried out complementation assay using miR-125b mimic. SupT1 cells were infected with VSV-G pseudotyped HIV-1-Luciferase reporter virus in the presence of cocaine. Thereafter, miR-125b mimic was transfected to these cells. As expected cocaine enhanced HIV-1 replication in these cells as reflected by increased luciferase activity (Fig. 7). This increase in HIV-1 replication by cocaine was abrogated by miR-125b mimic expression. These results demonstrate that miR-125b plays a direct role for cocaine-induced increase in HIV-1 replication.

### Cocaine Downregulates miR-125b in HIV-1 Infected CD4+ T Cells

**Figure 8:**
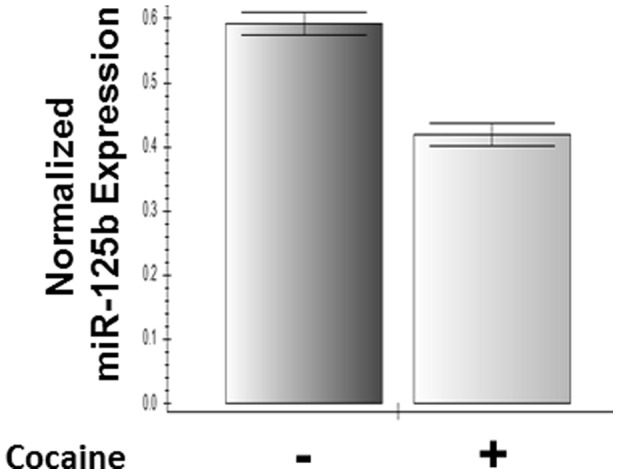
Cocaine downregulates miR-125b in HIV-1 infected CD4+ T cells. Activated primary CD4+ T cells were infected with HIV-1 LAI and treated with cocaine. Cocaine treatment downregulated miR-125b in infected primary CD4+ T cells as determined by real time PCR. Data are representative of three independent experiments conducted in triplicates.

To examine whether cocaine has the ability to downregulate miR-125b in HIV-1 infected cells, we infected primary CD4+ T cells with HIV-1 LAI and treated them with cocaine. We carried out RT-PCR experiment using RNA from these cells and data in Fig. 8 illustrate that upon cocaine treatment, miR-125b expression is downregulated in the infected cells.

### Cocaine Modulates miR-125b Promoter Driven Transcription

**Figure 9:**
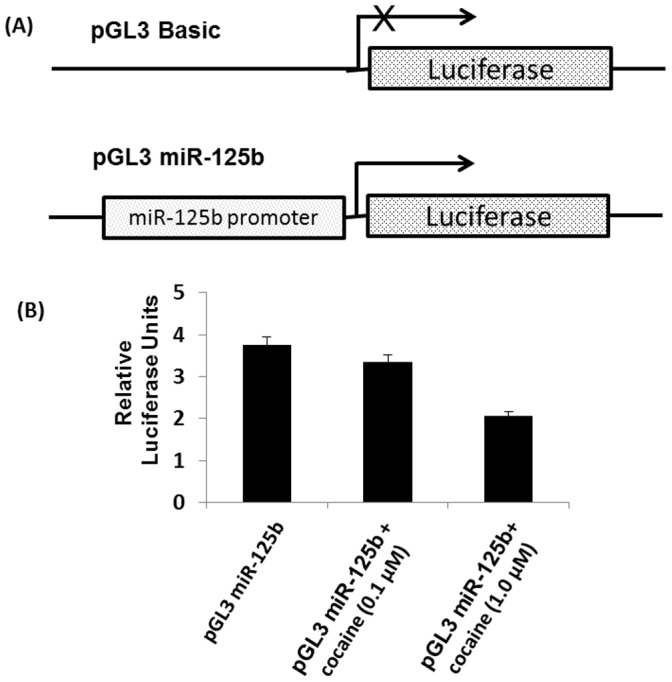
Cocaine regulates transcription of miR-125b. (A) A schematic representation of miR-125b promoter construct used. In this construct the miR-125b promoter drives the luciferase gene. (**B**) A construct containing miR-125b promoter driven luciferase gene or the vector control were transfected into 293T cells by Lipofectamine transfection. After 24 hrs, these cells were treated with cocaine (0.1 µM and 1 µM) for 4 hrs. Thereafter, cells were lysed and luciferase activity was measured by a luminometer. Cocaine treatment decreased miR-125b driven luciferase expression. Data are representative of three independent experiments conducted in triplicates.

To understand a mechanism by which cocaine downregulates miR-125b, we tested whether cocaine modulates the promoter activity of miR-125b. To test this, we transfected 293T cells with a pGL3 construct containing the miR-125b promoter that drives transcription of luciferase reporter (Fig. 9A). Thereafter, these cells were treated with cocaine at 0.1 µM and 1 µM concentrations. Upon cocaine treatment transcription of luciferase in these cells was decreased as measured by luciferase activity (Fig. 9B). These data sets suggest that cocaine regulate miR-125b expression by suppressing miR-125b transcription.

## Discussion

Cocaine serves as a cofactor for susceptibility to HIV infection [11]–[12] and AIDS progression [13]. In addition, epidemiological studies indicate that HIV positive cocaine users have lower CD4+ T cell counts and have a significant acceleration of decline of CD4+ T cells [9]–[13]. *In vitro* cocaine increases HIV-1 replication in cell culture models, peripheral blood mononuclear cells (PBMCs) [14]–[15] and enhances viral load in humanized mouse models [16]. Since CD4+ T cells are the primary targets for HIV-1 infection/replication *in vivo* [24], the goal of this study was to evaluate cocaine-induced enhancement of HIV-1 replication in primary CD4+ T cells [18]. Results presented here demonstrate for the first time that cocaine enhances HIV-1 replication in primary CD4+ T cells by downregulating the cellular anti-HIV miRNA miR-125b. These findings warrant a comprehensive understanding of cocaine-induced alterations in CD4+ T cell biology that will help us decipher the underlying mechanisms by which HIV-1 positive cocaine users have accelerated HIV-1 pathogenesis and AIDS-related death.

Although the mechanism by which cocaine enhances HIV replication remains unclear, there is strong evidence that cocaine modulates HIV-1 entry to the target cells [20]–[21]. HIV-1 entry is mediated by the binding of viral glycoproteins (HIV-1 gp120 and gp41) to CD4 and chemokine receptors (CCR5 and CXCR4) that leads to membrane fusion and release of viral core into the cytoplasm of target cells [36]–[38]. It has been proposed that cocaine enhances viral entry by inhibiting expression of HIV-suppressing chemokines in target cells [20]–[21]. These include regulated upon-activation T cell expressed and secreted (RANTES), macrophage inflammatory protein 1a (MIP-1a), and MIP-1b [22]. These molecules are known to inhibit binding/fusion/entry of the virus mediated by the HIV-1 envelope [22]. In addition, it has also been reported that cocaine enhances HIV-1 entry by upregulating the expression of entry co-receptors in the target cell [20]–[21]. These findings have been instrumental in depicting viral entry as the major target for cocaine induced enhancement of HIV-1 replication. In contrast to this accepted model, results presented in this study indicate that cocaine targets the post entry steps of HIV-1 life cycle since cocaine was added to the CD4+ T cells after infection (Fig. 1). This argument is further strengthened by the results that demonstrate cocaine-induced increased replication of VSV-G pseudotyped HIV-1 virions (Fig. 2). This is because pseudotyping allows HIV-1 to enter the target cell via endocytosis by abrogating CD4 and CCR5/CXCR4 receptor requirement for virus entry. It is important to point out that the post entry events of HIV-1 replication can be broadly categorized into reverse transcription, integration, transcription, translation, assembly and release steps [24]. Our microarray and real time PCR analysis demonstrated that cocaine downregulated the cellular anti-HIV miRNA miR-125b in primary CD4+ T cells (Fig. 3). Given that miR-125b has been reported to inhibit viral translation, these data corroborate our contention that cocaine targets post entry steps of HIV life cycle. In addition, our data illustrated that cocaine down-regulates miR-125b in HIV-1 infected CD4+ T cells.

miR-125b is a member of anti-HIV-1 miRNA family (including miR-28, miR-125b, miR-150, miR-223, and miR-382) that targets the 3′UTR of HIV-1 transcripts and inhibit viral translation, a post entry step. Our contention is also supported by the recent report that morphine downregulates the expression of the anti-HIV-1 miRNA in cultured human monocytes to enhance HIV-1 replication [44]. Therefore, based on these findings and available data, we propose that cocaine modulates both the entry and post-entry steps in the target cells to enhance HIV-1 replication. We believe cocaine enhances HIV-1 replication by targeting viral protein translation step in CD4+ T cells, since knock-down of miR-125b resulted in increased HIV-1 expression and overexpression of miR-125b inhibited HIV-1 expression (Fig. 5–6).

Although a body of recent literature suggests a multitude of regulatory functions of miR-125b in cell survival, differentiation and multiple malignancies (reviewed in [27]), the cellular targets of miR-125b in CD4+ T cells are not fully identified. miR-125b has been suggested to play a key role in maintaining the resting state of naïve CD4+ T cells since it has been proposed to regulate a network of genes in CD4+ T cells that are critical for its differentiation [28]. It has also been suggested that miR-125b and other anti-HIV-1 miRNAs may be responsible for inducing latency in naïve CD4+ T cells by inhibiting viral translation [29]. Very recently, it has been reported that miR-125b is downregulated in the PBMCs of HIV-1 infected individuals [31]. Intriguingly, downregulation of miR-125b has been implicated in higher level of viremia in these patients. There is increasing evidence that cellular miRNAs may negatively regulate of HIV-1 by either targeting HIV-1 mRNAs, and/or modulate expression of cellular factors [32]–[33]. A survey of literature and bioinformatics analysis did not identify known cellular targets such as RANTES, Sigma-1 receptor, MIP-1a/b, etc., that are known to play a role in cocaine induced enhancement of HIV-1 replication. Although we cannot exclude the indirect effects of miR-125b, it is likely that the observed effects of cocaine on HIV-1 replication is most likely due to direct targeting of HIV-1 genome by miR-125b. Therefore, our findings allude to novel mechanisms/pathways by which cocaine may enhance HIV-1 replication in CD4+ T cells. Most importantly, identifying the targets of miR-125b in CD4+ T cells will help us decipher the mechanisms/pathways targeted by cocaine. This knowledge will culminate in understanding the basis for increased HIV-1 pathogenesis among cocaine addicts and identifying potentially novel targets for future therapeutic intervention.

### Conclusions

This study demonstrates for the first time that cocaine enhances HIV-1 replication in primary CD4+ T cells by downregulating the antiviral innate immunity of cellular miRNA “miR-125b”. Since the accepted paradigm underlying cocaine-induced enhancement of HIV-1 replication is focused on viral entry, data presented here adds to the complexity of interplay between cocaine and HIV/AIDS by revealing involvement of HIV-1 post entry steps.
