# Correction: Cocaine Enhances HIV-1 Replication in CD4+ T Cells by Down-Regulating MiR-125b

**DOI:** 10.1371/journal.pone.0199338

**Published:** 2018-06-13

**Authors:** Chinmay K. Mantri, Jui Pandhare Dash, Jyoti Velamarti Mantri, Chandravanu Dash

The authors hereby provide some clarifications and corrections regarding the rationale, methods, and limitations of the microarray analysis in the study [1].

Select microRNAs were chosen for qPCR analysis in this study based on their anti-HIV activity [2].

In our original microarray analysis, two-channel microarrays were used to determine expression of miRNAs in treated and untreated cells. Even though every attempt was made to balance the channels during scanning, in these analyses, no dye-swap was performed. Despite the comparative reliability of the spike-ins, there is a possibility that the dye bias and background issues may confound the outcome of the array data. Therefore, we cannot exclude the prospect that the difference in miRNA expression is due to cocaine or to the dye channel. This may also lead to a broader effect on changes in miRNA expression not only in the treated samples.

The authors also want to clarify details as to the methods used for data normalization and processing and for statistical testing. The experimental details used in the microarray analysis involve labeling experiments following the Exiqon Hi-Power Labeling Kit (Cat#208035, lot#3000) protocol. The reactions were set up with the maximum available input in a 3ul volume between the 2 samples submitted for co-hybridization. The amounts used in each reaction were (in hyb order) 0.75ug, 0.75ug, and 0.5ug. Exiqon recommendations are from 0.25–1.0ug, using greater input with samples that are known to be low in miRNA. The Spike miRNA Kit, v2 (Cat#208041, lot#2002) was used in all the labeling reactions to check for target labeling efficiency and hybridization. The Cy5 /Cy3 labeled targets were combined per hyb order instructions, then mixed with 2x Hyb Buffer, heat denatured at 95°C for 2 minutes and quick-chilled on ice. The targets were loaded on an Agilent 1plex coverslip in an Agilent hyb chamber. The targets were then covered with the Exiqon arrays (Item # 20–1122; Lot#34001.03, 6th generation (product#208401-A Lot#6000) and the hybridization chamber was sealed. The hybridizations were performed per protocol specifications, for ~16–18 hours (17), 56C, 20 rpm in an Agilent Hybridization oven. Data were acquired with GENEPIX Pro v6.1. The data were analyzed on Agilent’s Feature Extraction software. Statistical analyses were performed using ANOVA and P values were calculated using Student’s t test. The error bars represent standard errors.

To the authors’ knowledge, the p values shown in Figure 3A were based on analysis of raw expression data provided by the genomics core. No corrections for multiple testing were done.

Despite the confounding nature of the microarray data, data shown in Figures 3–6 through our real-time analysis, knock down, and overexpression studies support the central hypothesis and conclusions of the study.

The microarray data from this study are deposited at GEO and available on the record GSE46398.

The fourth author’s name is incorrect. The correct name is: Chandravanu Dash. The correct citation is: Mantri CK, Pandhare Dash J, Mantri JV, Dash C (2012) Cocaine Enhances HIV-1 Replication in CD4+ T Cells by Down-Regulating MiR-125b. PLoS ONE 7(12): e51387. https://doi.org/10.1371/journal.pone.0051387
